# High-efficiency electrocatalytic nitrite reduction toward ammonia synthesis on CoP@TiO_2_ nanoribbon array

**DOI:** 10.1016/j.isci.2023.107100

**Published:** 2023-06-14

**Authors:** Xun He, Zixiao Li, Jie Yao, Kai Dong, Xiuhong Li, Long Hu, Shengjun Sun, Zhengwei Cai, Dongdong Zheng, Yongsong Luo, Binwu Ying, Mohamed S. Hamdy, Lisi Xie, Qian Liu, Xuping Sun

**Affiliations:** 1 Institute for Advanced Study, Chengdu University, Chengdu, Sichuan 610106, China; 2 Institute of Fundamental and Frontier Sciences, University of Electronic Science and Technology of China, Chengdu, Sichuan 610054, China; 3 College of Chemistry, Chemical Engineering and Materials Science, Shandong Normal University, Jinan, Shandong 250014, China; 4 Catalysis Research Group (CRG), Department of Chemistry, College of Science, King Khalid University, P.O. Box 9004, 61413 Abha, Saudi Arabia

**Keywords:** Electrochemistry, Materials chemistry

## Abstract

Electrochemical reduction of nitrite (NO_2_^−^) can satisfy the necessity for NO_2_^−^ contaminant removal and deliver a sustainable pathway for ammonia (NH_3_) generation. Its practical application yet requires highly efficient electrocatalysts to boost NH_3_ yield and Faradaic efficiency (FE). In this study, CoP nanoparticle-decorated TiO_2_ nanoribbon array on Ti plate (CoP@TiO_2_/TP) is verified as a high-efficiency electrocatalyst for the selective reduction of NO_2_^−^ to NH_3_. When measured in 0.1 M NaOH with NO_2_^−^, the freestanding CoP@TiO_2_/TP electrode delivers a large NH_3_ yield of 849.57 μmol h^−1^ cm^−2^ and a high FE of 97.01% with good stability. Remarkably, the subsequently fabricated Zn–NO_2_^−^ battery achieves a high power density of 1.24 mW cm^−2^ while delivering a NH_3_ yield of 714.40 μg h^−1^ cm^−2^.

## Highlights

• CoP@TiO_2_/TP acts as a superb NO_2_^−^RR electrocatalyst for NH_3_ synthesis
• It achieves a NH_3_ yield of 849.57 μmol h^−1^ cm^−2^ and a Faradaic efficiency of 97.01%
• It shows good stability for 12 h of bulk electrolysis and recycling tests
• The fabricated Zn–NO_2_^−^ battery achieves a NH_3_ yield of 714.40 μg h^−1^ cm^−2^

## Graphical abstract

**Figure:**
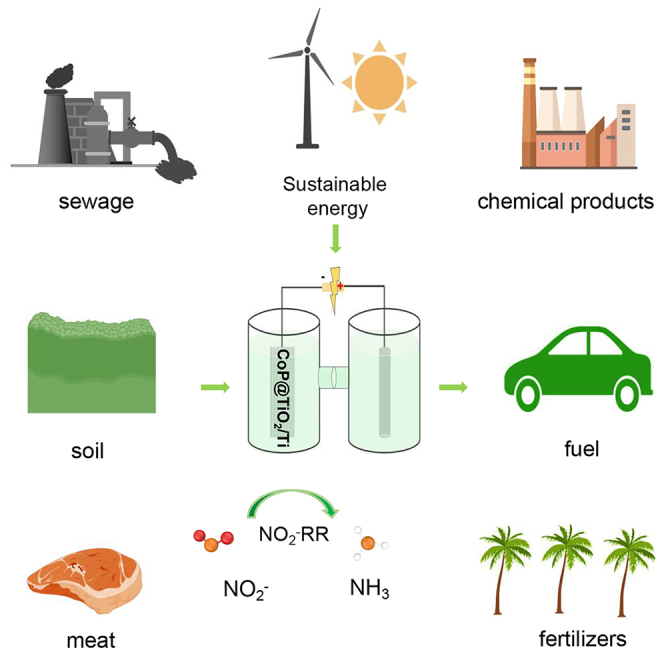

## Introduction

Ammonia (NH_3_) is a vital chemical feedstock in the manufacturing of fertilizers, explosives, rubber, etc., and is deemed as a fascinating next-generation energy supply source for non-carbon fuel cell.^1^^,^^2^^,^^3^^,^^4^ Presently, industrial massive synthesis of NH_3_ counts on the Haber-Bosch method, which yet suffers from numerous energy consumption and global carbon oxide emissions.^5^^,^^6^ In this regard, lots of effort have been focused on electrochemical nitrogen reduction reaction (NRR) in aqueous media, but NRR is a gas-liquid-solid reaction with low nitrogen solubility (6.8 × 10^−4^ M in water) that many catalysts are not ideal for nitrogen adsorption and cleavage and have low overpotential for hydrogen evolution reaction, which seriously hinders the activity and selectivity.^7^^,^^8^^,^^9^^,^^10^^,^^11^^,^^12^^,^^13^^,^^14^^,^^15^^,^^16^^,^^17^^,^^18^ Nitrite (NO_2_^−^), in contrast, is a highly water-soluble compound with weak N=O bond (204 kJ mol^−1^).^19^^,^^20^ It is not only generally found in soil and sewage but commonly applied in curing meat products, and its extreme accumulation poses environment and human health hazards.^21^^,^^22^ Encouragingly, electrochemical NO_2_^−^ reduction not only eliminates NO_2_^−^ pollutants but also yields NH_3_, but this process involves a six-electron transfer process that requires high-efficiency NO_2_^−^ reduction reaction (NO_2_^−^RR) catalysts to generate NH_3_.^23^^,^^24^

Precious metal-based catalysts are active toward NO_2_^−^RR, but their scarcity and high cost severely hinder their application.^25^^,^^26^^,^^27^^,^^28^ Earth-abundant and low-budget non-precious alternatives are therefore very attractive.^29^^,^^30^^,^^31^^,^^32^^,^^33^^,^^34^^,^^35^ In particular, CoP has attracted increasing interest for its high conductivity and operational persistence, as well as outstanding H-adsorbing ability for catalytic hydrogenation reactions,^36^^,^^37^ and has been confirmed to have NO_2_^−^RR activity.^38^^,^^39^ Recent studies have also verified that TiO_2_, which has the merits of being non-toxic, chemically stable, and structurally stable, is commonly applied to disperse highly reactive metal-based materials.^40^^,^^41^^,^^42^^,^^43^ TiO2 is active toward the NO_2_^−^RR, and its catalytic efficiency can be further improved by P or V doping.^19^^,^^44^^,^^45^ We thus believe that CoP@TiO_2_ composite can effectively catalyze the NO_2_^−^-to-NH_3_ conversion, which however has not been addressed so far.

Herein, we present our recent experiment results that CoP nanoparticle-decorated TiO_2_ nanoribbon array supported on Ti plate (CoP@TiO_2_/TP) serves as a superb NO_2_^−^RR catalyst for ambient NH_3_ electrosynthesis with excellent selectivity. When tested in alkaline environments, CoP@TiO_2_/TP attains an extraordinary NH_3_ yield of 849.57 μmol h^−1^ cm^−2^ and a high NH_3_ Faradaic efficiency (FE) of 97.01%. Furthermore, we demonstrated a Zn–NO_2_^–^ battery with CoP@TiO_2_/TP cathode has high power density as well as generating satisfying NH_3_ yield.

## Results and discussion

**Figure 1:**
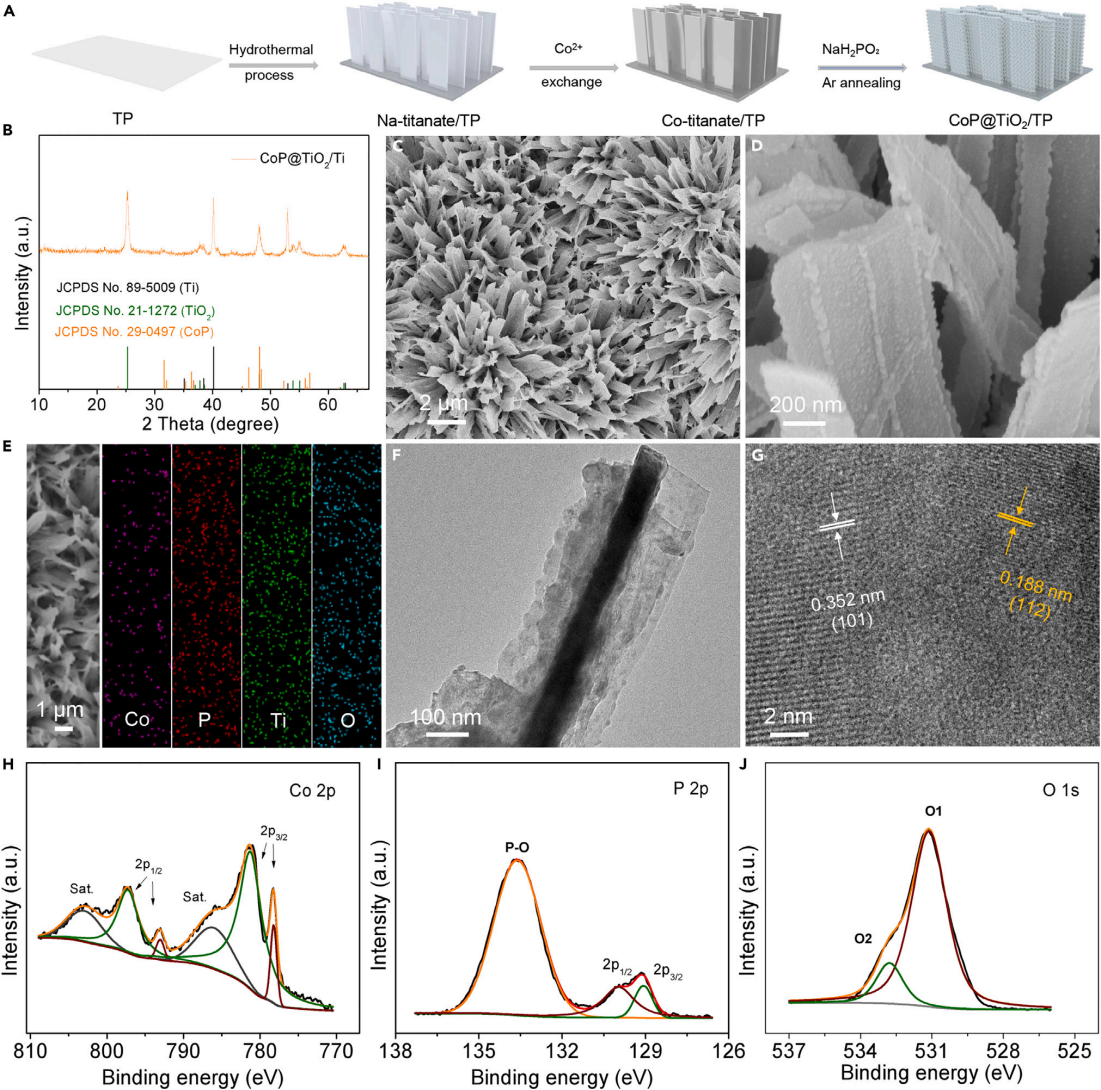
Structural characteristics of CoP@TiO_2_/TP (A) Schematic diagram of the fabrication process of CoP@TiO_2_/TP. (B) XRD pattern of CoP@TiO_2_/TP. (C) Low- and (D) high-magnification SEM images of CoP@TiO_2_/TP. (E) SEM and corresponding EDX elemental mapping images of CoP@TiO_2_. (F) TEM and (G) HRTEM images of CoP@TiO_2_. (H–J) High-resolution XPS spectra of CoP@TiO_2_ in the (H) Co 2p, (I) P 2p, and (J) O 1s regions.

As depicted in Figure 1A, CoP@TiO_2_/TP was fabricated via hydrothermal process, Co^2+^ exchange, and Ar annealing phosphorylation. Figures 1B and S1 exhibit the X-ray diffraction pattern of CoP@TiO_2_/TP and TiO_2_/TP, which both display the diffraction peak features of TiO_2_ (JCPDS No. 21–1272) and Ti (JCPDS No. 89–5009), while the remaining peaks of CoP@TiO_2_/TP are assigned to CoP (JCPDS No. 29–0497). The SEM images in Figures S2 and S3 exhibit that TP is fully covered by TiO_2_ nanoribbon array. And SEM (Figures 1C and 1D) and transmission electron microscopy (TEM) (Figure 1F) images indicate that CoP nanoparticle precipitated from the interlayer of TiO_2_ during the phosphorylation reaction and were embedded on the surface of TiO_2_ nanoribbon array. The SEM and corresponding energy-dispersive X-ray elemental mapping images (Figure 1E) of CoP@TiO_2_ reveal the even element distribution of Co, P, Ti, and O, and the mass percentage of CoP in CoP@TiO_2_ is approximately 28.92% (Figure S4). Furthermore, the high-resolution TEM image of CoP@TiO_2_ (Figure 1G) validates lattice spacings of 0.188 and 0.352 nm, ascribed to the (112) and (101) crystal surfaces of CoP and TiO_2_, respectively. It is therefore reasonable to infer we have successfully prepared CoP nanoparticle-decorated TiO_2_ nanoribbon array supported on TP. Besides that, the X-ray photoelectron spectroscopy (XPS) spectrum was applied to study the surface chemical compositions of CoP@TiO_2_. As shown in Figure 1H, the XPS spectrum of CoP@TiO_2_ in Co 2p region is divided into six peaks. The peaks at the binding energies (BEs) of 797.25 and 793.04 eV are assigned to Co 2p_1/2_, and the peaks at the BEs of 781.25 and 778.21 eV match with Co 2p_3/2_, while the peaks at the BEs of 802.94 and 785.99 eV are assigned to two satellites (defined as “Sat.”).^37^^,^^46^ In the P 2p region (Figure 1I), three peaks at the BEs of 133.61, 129.07, and 129.96 eV are associated with P-O, P 2p_1/2_, and P 2p_3/2_, respectively.^37^^,^^47^ In the Ti 2p region (Figure S5), two peaks at the BEs of 458.87 and 464.69 eV are attributed to Ti 2p_3/2_ and Ti 2p_1/2_, respectively.^48^ Besides, the O 1s region spectrum was fitted to two peaks, located at the BEs of 531.12 and 532.78 eV, ascribed to the metal-oxygen bond (O_1_) and the lattice oxygen (O_2_), respectively (Figure 1J).^48^^,^^49^

**Figure 2:**
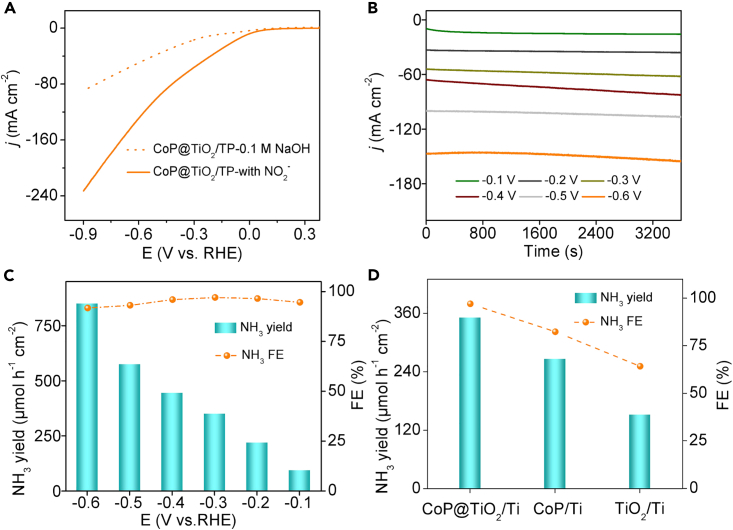
Electrochemical NO_2_^−^RR tests (A) LSV curves of CoP@TiO_2_/TP in 0.1 M NaOH with/without 0.1 M NO_2_^−^. (B) CA curves of CoP@TiO_2_/TP at various potentials. (C) NH_3_ yields and FEs of CoP@TiO_2_/TP at various potentials. (D) Comparison of NH_3_ yield and FE of CoP@TiO_2_/TP, CoP/TP, and TiO_2_/TP at −0.3 V.

The electrocatalytic activity of NO_2_^−^RR was evaluated in 0.1 M NaOH electrolyte with 0.1 M NO_2_^−^. The indophenol blue and the Watt and Crisp method were used to count NH_3_ and the potential by-product of N_2_H_4_ (Figures S6 and S7), respectively. Figures 2A and S8 present the linear scanning voltammetry (LSV) curves of CoP@TiO_2_/TP, CoP/TP, and TiO_2_/TP. It is obvious that CoP@TiO_2_/TP delivers a larger current density (*j*) once NO_2_^−^ is added, which reveals that CoP@TiO_2_/TP can catalyze the reduction of NO_2_^−^ effectively. Comparatively, TiO_2_/TP and CoP/TP with the presence of NO_2_^−^ show a lower *j*. We then performed chronoamperometry tests to investigate NH_3_ yields and FEs from −0.1 to −0.6 V (Figure 2B). It unveils more NH_3_ is formed as the cathodic potential rises (Figure S9). Notably, the FEs of CoP@TiO_2_/TP are high at each potential (over 90%) and the highest value of 97.01% occurred at −0.3 V with a corresponding NH_3_ yield of 350.87 μmol h^−1^ cm^−2^. And such CoP@TiO_2_/TP achieved a superb NH_3_ yield of 849.57 μmol h^−1^ cm^−2^ at −0.6 V. It is notable that the electrocatalytic activity of CoP@TiO_2_/TP exceeds that of most the already reported NO_2_^−^RR catalysts mentioned in Table S1. Synchronously, the NO_2_^−^-to-NH_3_ transformation of CoP/TP and TiO_2_/TP was measured at −0.3 V. As shown in Figure 2D, CoP@TiO_2_/TP obviously showed better electrocatalytic activity than that of CoP/TP (82.3%, 266.15 μmol h^−1^ cm^−2^) and TiO_2_/TP (64.2%, 151.46 μmol h^−1^ cm^−2^). The outstanding NH_3_-producing ability of CoP@TiO_2_/TP is attributed to two main factors. Firstly, the self-supported CoP@TiO_2_/TP electrode eliminates the need for a polymer binder, thus enhancing electrode dynamics. Secondly, the readily available TiO_2_ array with its unique nanoribbon-like structure provides a large specific surface area that improves the dispersibility of CoP nanoparticle and prevents its agglomeration, thus enhancing the adsorption ability of NO_2_^−^.

**Figure 3:**
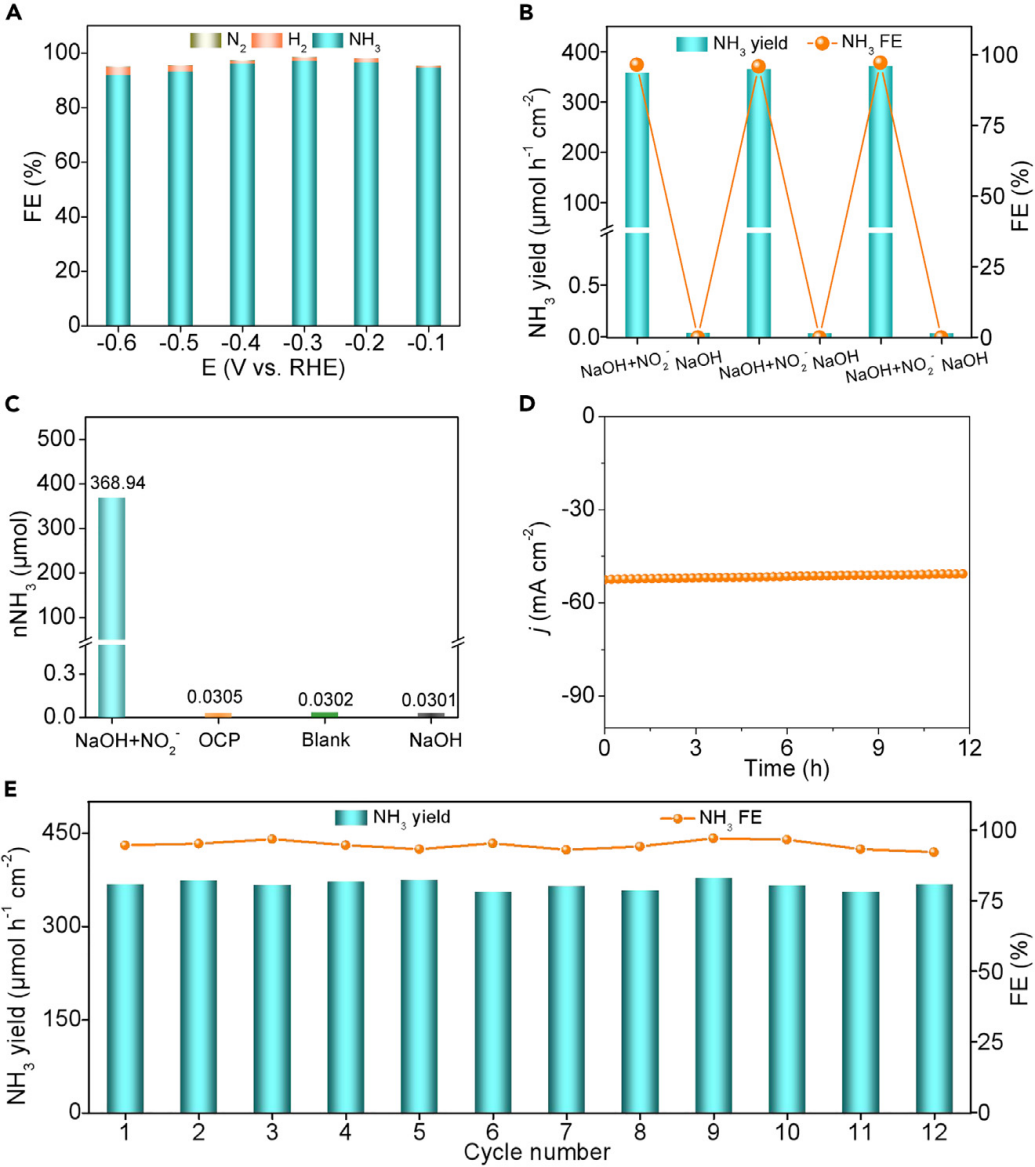
By-product analysis and stability tests of CoP@TiO_2_/TP toward NO_2_^−^RR (A) FEs of H_2_, N_2_, and NH_3_ for CoP@TiO_2_/TP at different potentials. (B) NH_3_ yields and FEs of CoP@TiO_2_/TP during the alternating cycling tests. (C) NO_2_^−^RR performance of CoP@TiO_2_/TP under different test conditions. (D) Time-dependent current density curve during 12-h electrolysis of CoP@TiO_2_/TP at −0.3 V. (E) Recycling tests of CoP@TiO_2_/TP at −0.3 V.

The catalytic process of CoP@TiO_2_/TP was following evaluated by identifying diverse by-products (H_2_, N_2_H_4_, and N_2_) in the complex pathway from NO_2_^−^ to NH_3_. Of note, we found that CoP@TiO_2_/TP did not produce N_2_H_4_ toward NO_2_^−^RR process (Figure S10). And the partial current density and FEs of the gas-phase H_2_ and N_2_ at the entire potential window were nearly negligible (Figures S11 and 3A), affirming great NO_2_^−^RR selectivity of CoP@TiO_2_/TP toward NH_3_ synthesis. We then performed alternating electrolysis at −0.3 V between NO_2_^−^-containing and NO_2_^−^-free solution for 6 cycles. It is obvious that NH_3_ was yielded only in the solution containing NO_2_^−^ (Figure 3B). And it can be further seen from Figures 3C and S12 that extremely small quantity of NH_3_ (all less than 0.04) was generated by electrolysis for 1 h in open circuit potential, blank solution, and NO_2_^−^-free NaOH electrolyte, which uncovers ammonia just derived from CoP@TiO_2_/TP, eliminating electrolyte and equipment interferences. Besides, we confirmed the long-lasting tolerance of CoP@TiO_2_/TP by electrolysis at −0.3 V for 12 h (*j* decreased by only 2%) (Figure 3D). Also, the NH_3_ yield and FE did not change much for 12 electrolysis cycles (Figures 3E and S13), indicating the outstanding repeatability of CoP@TiO_2_/TP for the ambient electroreduction of NO_2_^−^ to NH_3_. Significantly, the LSV curve (Figure S14), composition (Figure S15), and morphology (Figure S16) of CoP@TiO_2_/TP remain almost identical even after 12-h electrolysis. Those suggest the exceptional stability of CoP@TiO_2_/TP for NH_3_ generation by NO_2_^−^RR under working conditions.

**Figure 4:**
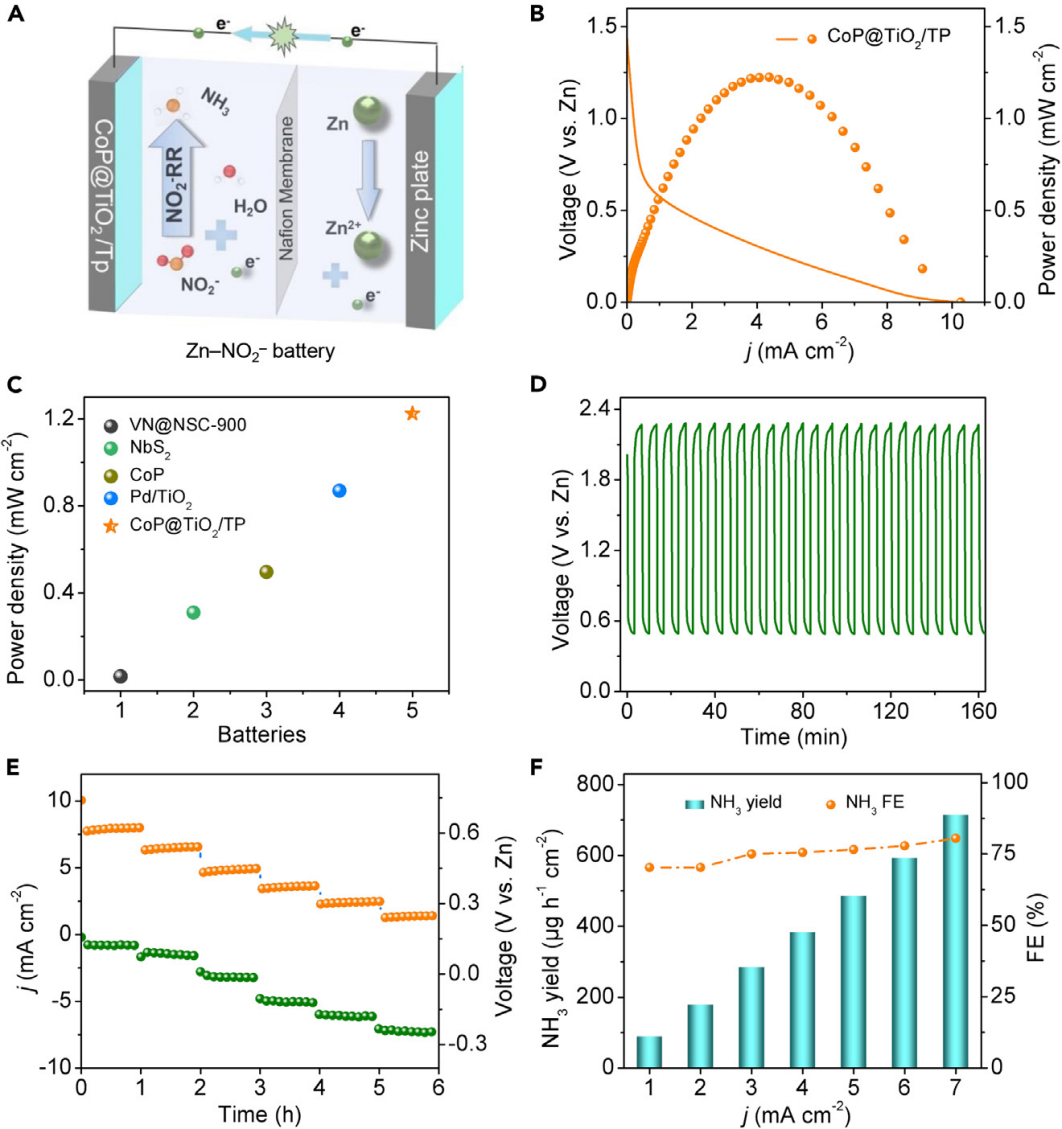
Zn–NO_2_^–^ battery with CoP@TiO_2_/TP cathode (A) Schematic illustration of the Zn–NO_2_^–^ battery. (B) Discharge curve and the resultant power density curve of the battery. (C) Comparison of power density for the current Zn–NO_2_^–^ battery with CoP@TiO_2_/TP as the cathode with the reported Zn–N_2_, Zn–NO, and Zn–NO_3_^–^ batteries. (D) Charge–discharge voltage profiles of the Zn–NO_2_^–^ battery at 2 mA cm^−2^. (E) Discharging tests at various current densities. (F) NH_3_ yields and FEs at different current densities.

Zn–NO_2_^–^ battery is capable of releasing energy with a theoretical voltage of 1.59 V and can provide a high power density of 964 Wh kg^−1^ while producing value-added NH_3_.^34^ Based on the previous analysis that CoP@TiO_2_/TP has been verified as a high-efficiency NO_2_^−^RR catalyst toward NH_3_ synthesis, we thus assembled the CoP@TiO_2_/TP-based Zn–NO_2_^–^ battery (Figure 4A). The performance of the fabricated battery was initially evaluated by a discharge curve, which showed an increase in output *j* when the cathode potential became more negative, and reached the maximum power density of 1.22 mW cm^−2^ (Figure 4B), higher than the reported aqueous Zn–N_2_, Zn–NO, and Zn–NO_3_^–^ batteries (Figure 4C).^47^^,^^50^^,^^51^^,^^52^ As exhibited in Figure 4D, the charge/discharge voltage profiles of such battery at 2 mA cm^−2^ displayed only slight deviations, which confirm the potential rechargeability of our battery. Besides, Figure 4E presents the discharging curves of the fabricated battery with various *j* for 1 h and the *j* increased gradually from 1 mA cm^−2^, reaching 7 mA cm^−2^ at approximate 0.25 V vs. Zn^2+^/Zn, demonstrating superior electrochemical performance and long-lasting stability. The NH_3_ yields and FEs of the CoP@TiO_2_/TP-based Zn–NO_2_^–^ battery were next measured as shown in Figure 4F. As expected, the FEs of NH_3_ production were appealing at various *j* and it shows a high FE of 80.45% with a NH_3_ yield of 714.4 μg h^−1^ cm^−2^ at a *j* of 7 mA cm^−2^. Therefore, a NO_2_^−^-containing energy conversion device involving NO_2_^−^RR is potential for applications.

### Conclusions

In summary, CoP@TiO_2_/TP is experimentally proved to be a high-efficiency NO_2_^−^RR electrocatalyst for NH_3_ production under ambient conductions, which is capable of yielding a large NH_3_ yield of 849.57 μmol h^−1^ cm^−2^ and a high FE of 97.01% with a long electrolytic durability. Impressively, the fabricated Zn–NO_2_^–^ battery obtains a remarkable power density of 1.22 mW cm^−2^ with a large NH_3_ yield of 714.4 μg h^−1^ cm^−2^ by utilizing CoP@TiO_2_/TP as a cathode, and it shows potential rechargeability. This work provides us with an earth-abundant catalyst material for ambient NH_3_ electrosynthesis and other applications.

### Limitations of the study

A CoP@TiO_2_/TP-based Zn–NO_2_^–^ battery presents a “killing three birds with one stone” strategy, providing energy supply, ammonia generation, and removal of pollutants. At the moment, however, it does not seem to be a good battery or ammonia synthesis device, hindering by its low ammonia yield and power density. In the future, research efforts will focus on developing cathode materials that can produce high ammonia yield and power density, as well as investigating the reactions that occur on the cathode during charging.

## STAR★Methods

### Key resources table

**Table:** 

REAGENT or RESOURCE	SOURCE	IDENTIFIER
**Chemicals, peptides, and recombinant proteins**		

Co(NO_3_)_2_·6H_2_O	Aladdin Co., Ltd.	10026-22-9
NaH_2_PO_2_	Aladdin Co., Ltd.	7681-53-0
NaNO_2_	Aladdin Co., Ltd.	7632-00-0
NH_4_Cl	Aladdin Co., Ltd.	12125-02-9
NaOH	Aladdin Co., Ltd.	1310-73-2
C_7_H_5_NaO_3_	Aladdin Co., Ltd.	54-21-7
C_6_H_5_Na_3_O_7_·2H_2_O	Aladdin Co., Ltd.	6132-04-3
C_9_H_11_NO	Aladdin Co., Ltd.	100-10-7
C_5_FeN_6_Na_2_O·2H_2_O	Aladdin Co., Ltd.	13755-38-9
NaClO	Aladdin Co., Ltd.	7681-52-9
H_2_SO_4_	Beijing Chemical Corporation	7664-93-9
H_2_O_2_	Beijing Chemical Corporation	7722-84-1
N_2_H_4_·H_2_O	Beijing Chemical Corporation	7803-57-8
HCl	Beijing Chemical Corporation	7647-01-0
C_2_H_5_OH	Beijing Chemical Corporation	64-17-5
Ti plate	Qingyuan Metal Materials Co., Ltd.	/

### Resource availability

#### Lead contact

Further information and requests for resources should be directed to and will be fulfilled by the lead contact, Dr. Xuping Sun (xpsun@uestc.edu.cn).

#### Materials availability

This study did not generate new unique reagents. All chemicals were obtained from commercial resources and used as received.

### Method details

#### Synthesis of CoP@TiO_2_/TP

To synthesize CoP@TiO_2_/TP, many pieces TP measuring 2.0 × 3.0 cm^2^ were sonicated in HCl, C_2_H_5_OH, and water for 10 min each. The pretreated TP were then placed in a Teflon-lined autoclave containing 5 M NaOH solutions and heated at 180°C for 24 h to obtain Na_2_Ti_2_O_5_/TP. The resulting Na_2_Ti_2_O_5_/TP was then immersed in 0.1 M Co(NO_3_)_2_ for 1 h to replace Na^+^ with Co^2+^. After washing with water and drying, the obtained CoTi_2_O_5_/TP was annealed with NaH_2_PO_2_ at 500°C for 1 h under an Ar atmosphere, resulting in the final product, CoP@TiO_2_/TP. For comparison, TiO_2_/TP was also synthesized using the same process as CoP@TiO_2_/TP, but the Na_2_Ti_2_O_5_/TP was immersed in diluted HCl to exchange Na^+^ to H^+^.

#### Characterizations

X-ray diffractometer (XRD) loaded a Cu Kα radiation target (40 kV, 30 mA) (SHIMADZU, Japan), scanning electron microscope (SEM) with 5 kV acceleration voltage (ZEISS, Germany), transmission electron microscopy (TEM) with a Zeiss Libra 200FE, and X-ray photoelectron spectroscopy (XPS) (ESCALAB 250 Xi) were applied to study the composition and morphology of the prepared CoP@TiO_2_ and TiO_2_. Gas chromatography (GC) (Shimadzu GC-2014C) was used to detect gaseous products. Ultraviolet-visible spectrophotometer (UV–vis) was applied to measure absorbance (SHIMADZU UV-1800).

#### Electrochemical measurements

Electrochemical tests were conducted in a H-type cell separated by a Nafion 117 membrane using a CHI 760E electrochemical workstation (Shanghai, Chenhua). The electrolyte solution (30 mL) was Ar-saturated 0.1 M NaOH with and without NO_2_^−^ (NaNO_2_), with CoP@TiO_2_/TP (0.5 × 0.5 cm^2^), graphite rod, and Hg/HgO as the working electrode, counter electrode, and reference electrode, respectively. To conform to the Nernst equation, all potentials were converted into the potential of the reversible hydrogen electrode (RHE) (*E*_*RHE*_ = *E*_*Hg/HgO*_ + 0.059 × pH + 0.098 V). Linear sweep voltammetry (LSV) curves were tested using the CHI 760E with a scan rate of 5 mV^−1^.

To determine the NH_3_ concentration in the solution, colorimetry was used (the obtained electrolyte was diluted 40 times) via the indophenol blue method. Specifically, 2 mL of the solution after the reaction was mixed with 2 mL of 1 M NaOH coloring solution containing 5% C_7_H_5_NaO_3_ and 5% C_6_H_5_Na_3_O_7_·2H_2_O. Then, 1 mL of oxidizing solution of 0.05 M NaClO and 0.2 mL of catalyst solution of C_5_FeN_6_Na_2_O (1 wt%) were added to the above solution. After standing in the dark for 2h, the UV–vis absorption spectra were measured, and the NH_3_ concentration was identified using the absorbance at a wavelength of 655 nm. The concentration-absorbance curve was calibrated using the standard NH_4_Cl solution with NH_3_ concentrations of 0, 0.2, 0.5, 1.0, 2.0, and 5.0 ppm in 0.1 M NaOH solution. The fitting curve (0.3541 x+0.00875, R^2^=0.9993) showed a good linear relation of absorbance value with NH_3_ concentration.

To estimate N_2_H_4_, the Watt and Crisp method was used. The color reagent was a solution of 18.15 mg/mL of C_9_H_11_NO in the mixed solvent of HCl and C_2_H_5_OH (V/V: 1/10). In detail, 2 mL of electrolyte was added to 2 mL of the color reagent for 15 min under stirring. The absorbance of such solution was measured to quantify the hydrazine yields by the standard curve of hydrazine (y = 0.68479 x + 0.10146, R^2^=0.9993).

Determination of NH_3_ yield and FE:

$$FE=\frac{nCVF}{MQ}$$

$$NH_{3}\;yield=\frac{CV}{17tA}$$

Here, n represents the number of electrons transferred during NO_2_^–^RR, C represents the concentration of products, V represents the volume of the cathodic electrolyte (35 mL), F is the Faradaic constant (96500 C mol^-1^), M is the molar mass of products, Q is the total quantity of applied electricity, t is the electrolysis time, and A is the geometric area of the working electrode (0.5 × 0.5 cm^2^). The partial current densities in Figure S11, one can multiply the average current density at each potential with the FE of each reduction product.

## Data Availability

• Data reported in this paper will be shared by the lead contact upon reasonable request. • This study does not report any original code. • Any additional information required to reanalyze the data reported in this paper is available from the lead contact upon reasonable request.
