# Supplementary material for: High-efficiency electrocatalytic nitrite reduction toward ammonia synthesis on CoP@TiO2 nanoribbon array
Source: iScience. 2023 Jun 14;26(7):107100. doi: 10.1016/j.isci.2023.107100 (PMC10329173; doi:10.1016/j.isci.2023.107100)
Supplement: Document S1. Figures S1–S17 and Table S1 [file mmc1.pdf]

iScience, Volume *26*

Supplemental information

High-efficiency electrocatalytic nitrite reduction toward ammonia synthesis on $CoP@TiO_2$ nanoribbon array

Xun He, Zixiao Li, Jie Yao, Kai Dong, Xiuhong Li, Long Hu, Shengjun Sun, Zhengwei Cai, Dongdong Zheng, Yongsong Luo, Binwu Ying, Mohamed S. Hamdy, Lisi Xie, Qian Liu, and Xuping Sun

Supplemental information

High-efficiency electrocatalytic nitrite reduction toward ammonia synthesis on $CoP@TiO_2$ nanoribbon array

Xun He,[1,2] Zixiao Li,[2] Jie Yao,[2] Kai Dong,[2] Xiuhong Li,[2] Long Hu,[2] Shengjun Sun,[3] Zhengwei Cai,[3] Dongdong Zheng,[3] Yongsong Luo,[2] Binwu Ying,[2] Mohamed S. Hamdy,[4] Lisi Xie,[1] Qian Liu,[1*] and Xuping Sun[2,3,5*]

[1]Institute for Advanced Study, Chengdu University, Chengdu 610106, Sichuan, China

[2]Institute of Fundamental and Frontier Sciences, University of Electronic Science and Technology of China, Chengdu 610054, Sichuan, China

[3]College of Chemistry, Chemical Engineering and Materials Science, Shandong Normal University, Jinan 250014, Shandong, China

[4]Catalysis Research Group (CRG), Department of Chemistry, College of Science, King Khalid University, P.O. Box 9004, 61413 Abha, Saudi Arabia

[5]Lead contact

*Correspondence: liuqian@cdu.edu.cn (Q.L.), xpsun@uestc.edu.cn (X.S.)

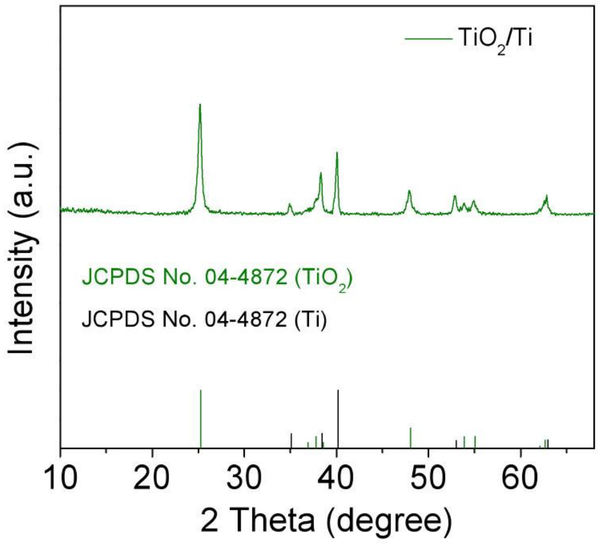

Figure S1. XRD pattern of TiO_2/TP, related to Figure 1.

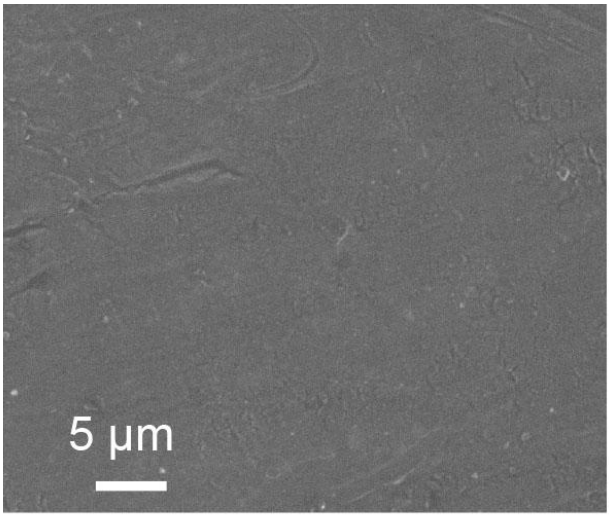

Figure S2. SEM image of TP, related to Figure 1.

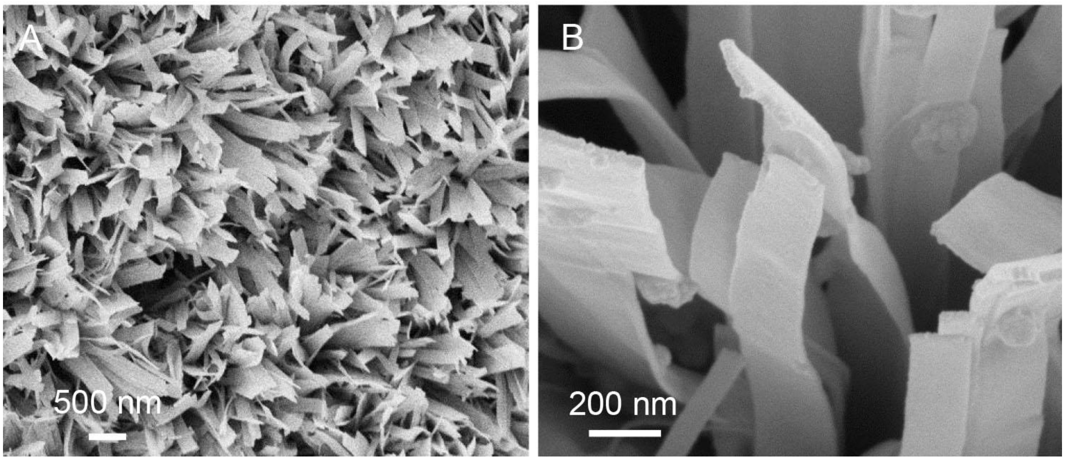

Figure S3. SEM images of TiO_2/TP, related to Figure 1.

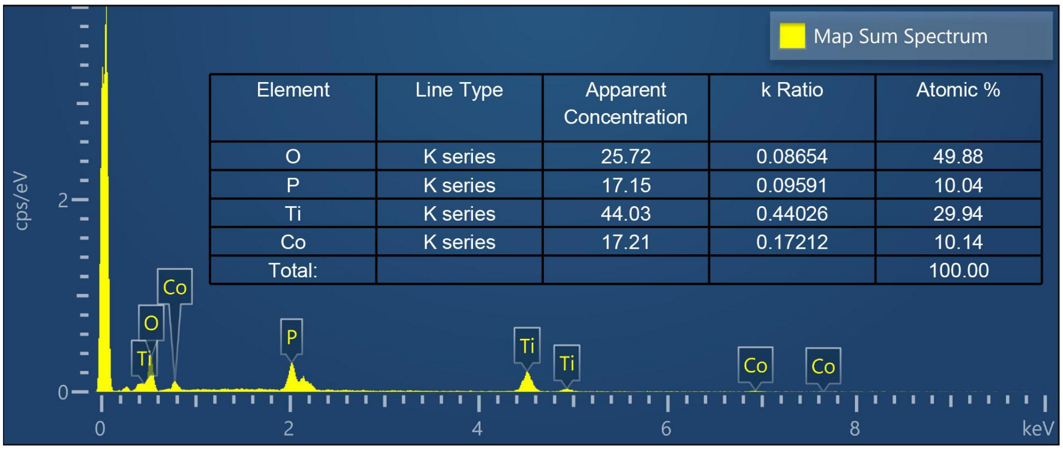

Element	Line Type	Apparent Concentration	k Ratio	Atomic %
O	K series	25.72	0.08654	49.88
P	K series	17.15	0.09591	10.04
Ti	K series	44.03	0.44026	29.94
Co	K series	17.21	0.17212	10.14
Total:				100.00

Figure S4. EDX spectrum of CoP@TiO_2, related to Figure 1.

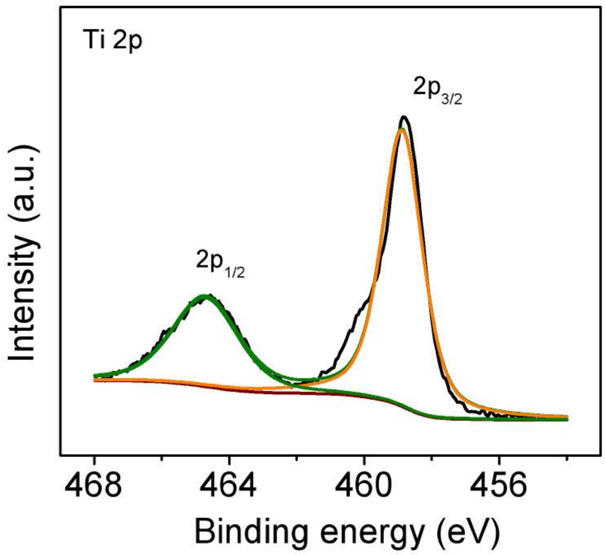

Figure S5. XPS spectrum of $CoP@TiO_2$ in the Ti 2p region, related to Figure 1.

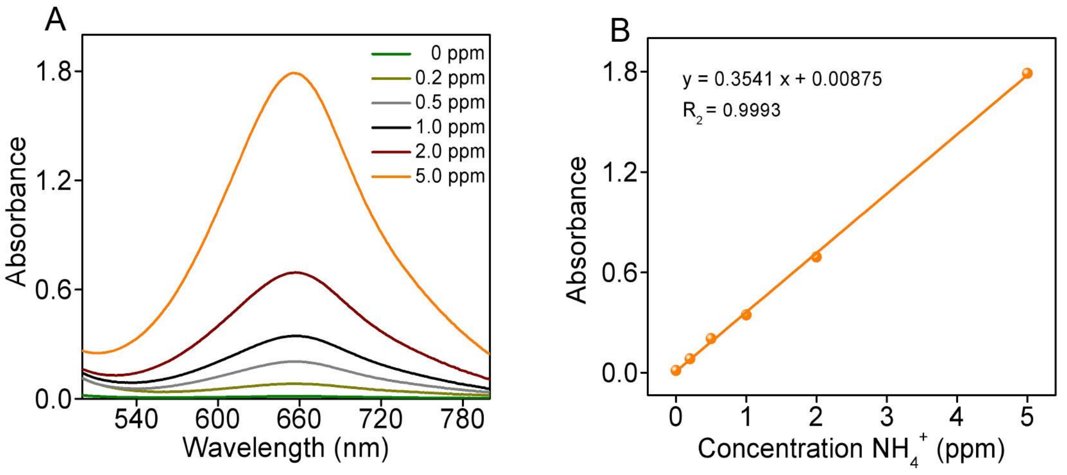

Figure S6. (a) UV–vis absorption spectra and (b) corresponding calibration curve for calculation of NH_4^+ concentration, related to STAR Methods and Figure 2.

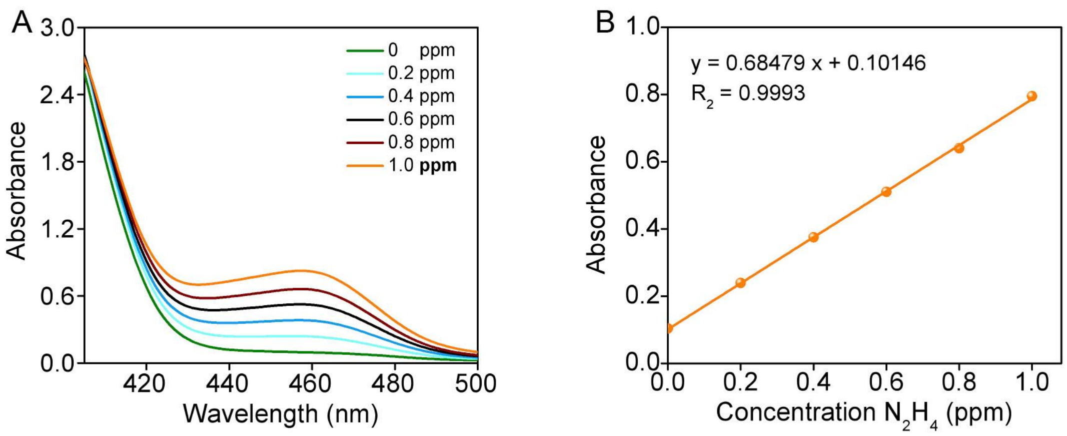

Figure S7. (a) UV–vis absorption spectra and (b) corresponding calibration curve for calculation of N_2H_4 concentration, related to STAR Methods and Figure 3.

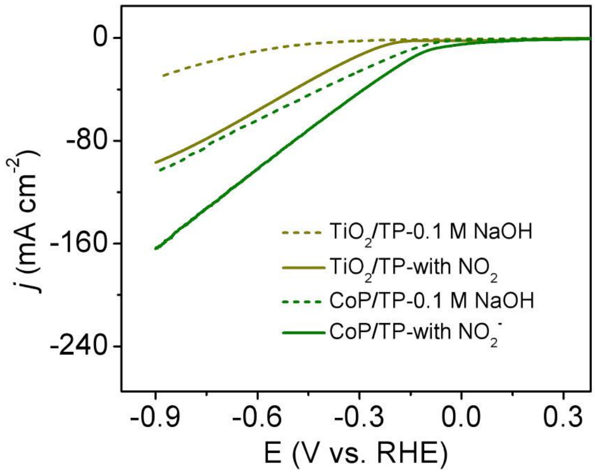

Figure S8. LSV curves of CoP/TP and TiO_2/TP in 0.1 M NaOH with/without 0.1 M NO_2^-, related to Figure 2.

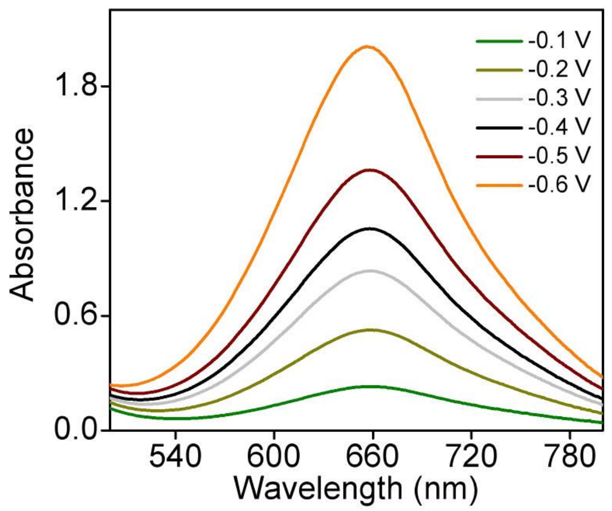

Figure S9. UV–vis absorption spectra of CoP@TiO_2/TP for the NO_2^-RR at different applied potentials, related to Figure 2.

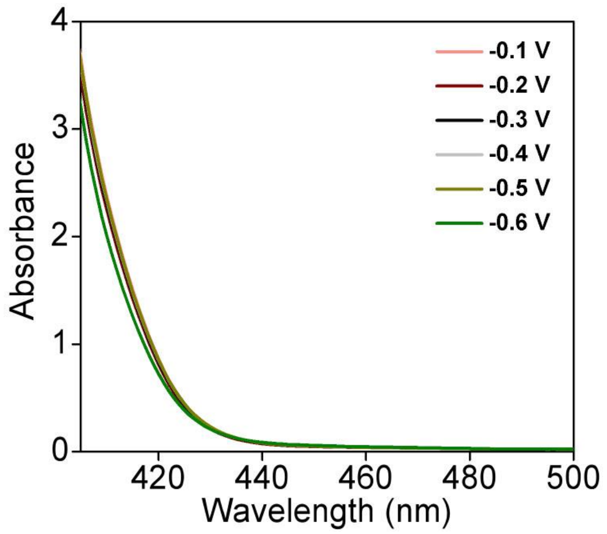

Figure S10. UV–vis absorption spectra of N_2H_4 for CoP@TiO_2/TP at different given potentials, related to Figure 3.

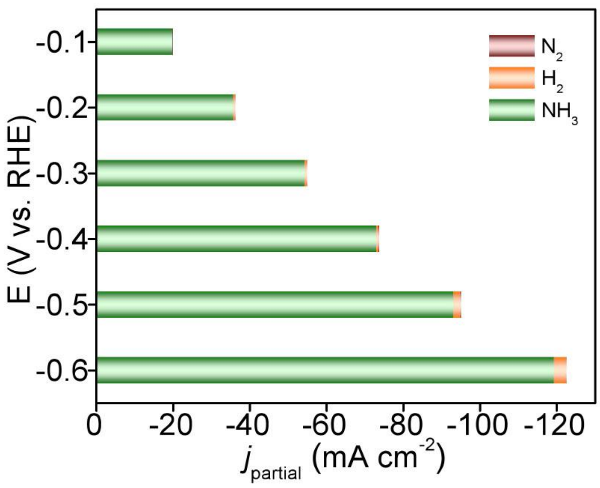

Figure S11. The partial current density ($j_{partial}$) of NH_3, H_2, and N_2 for CoP@TiO_2/TP, related to Figure 3.

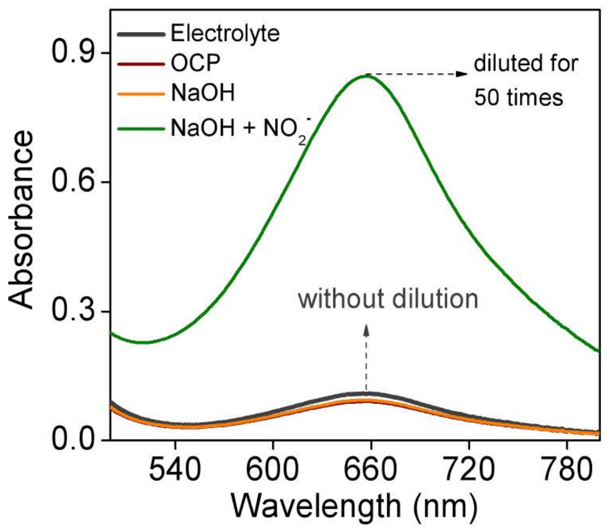

Figure S12. UV-vis absorption spectra of $CoP@TiO_2/TP$ under different test conditions, related to Figure 3.

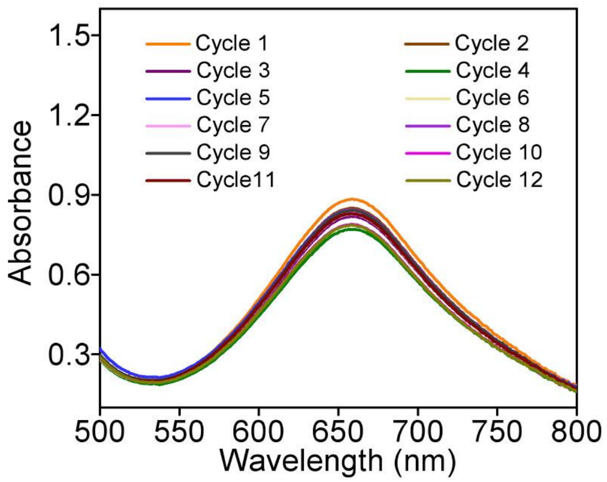

Figure S13. UV–vis spectra of CoP@TiO_2/TP for electrogenerated NH_3 during cycling tests at –0.3 V in 0.1 M NaOH with 0.1 M NO_2^-, related to Figure 3.

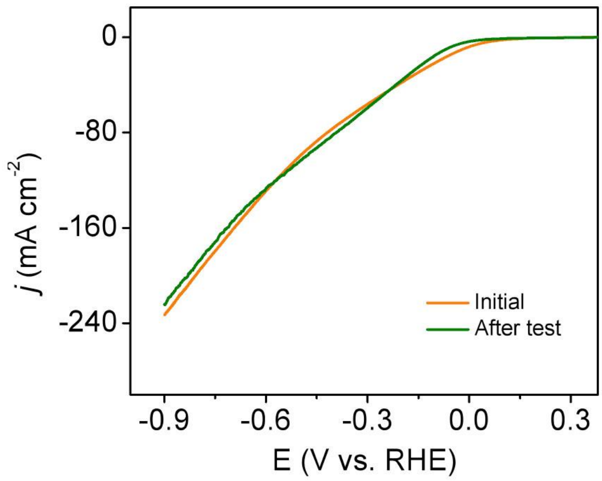

Figure S14. LSV curves of CoP@TiO_2/TP before and after long-term electrolysis, related to Figure 3.

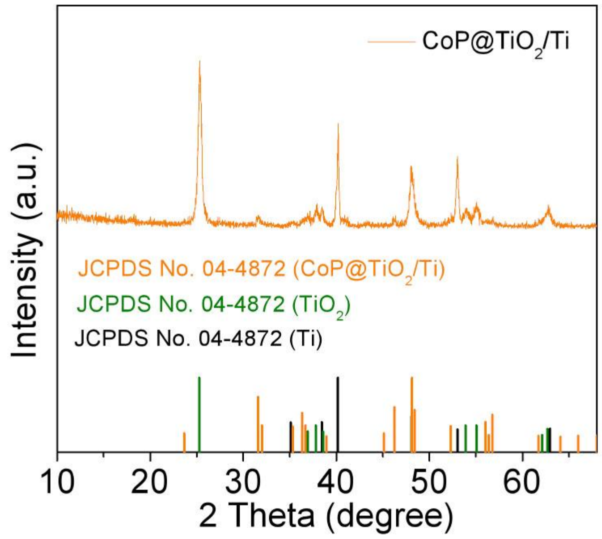

Figure S15. XRD pattern of $CoP@TiO_2/TP$ after long-term electrolysis, related to Figure 3.

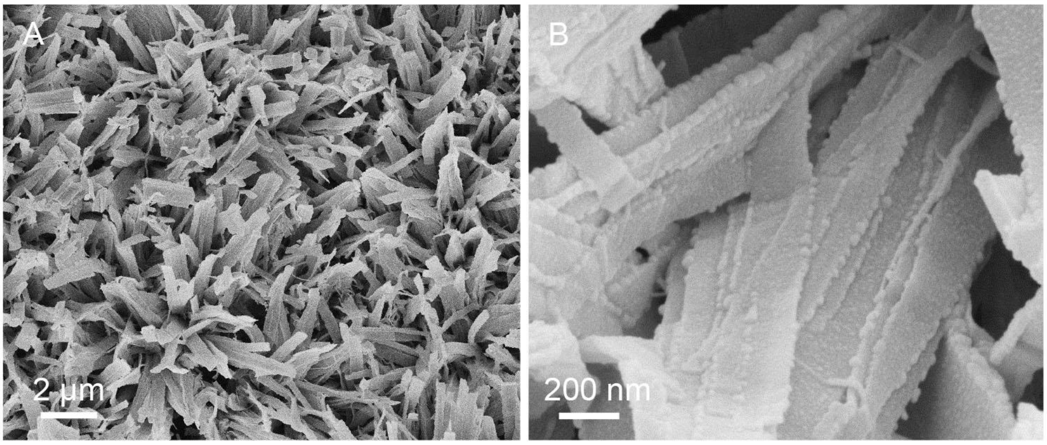

Figure S16. SEM images of CoP@TiO_2/TP after long-term electrolysis, related to Figure 3.

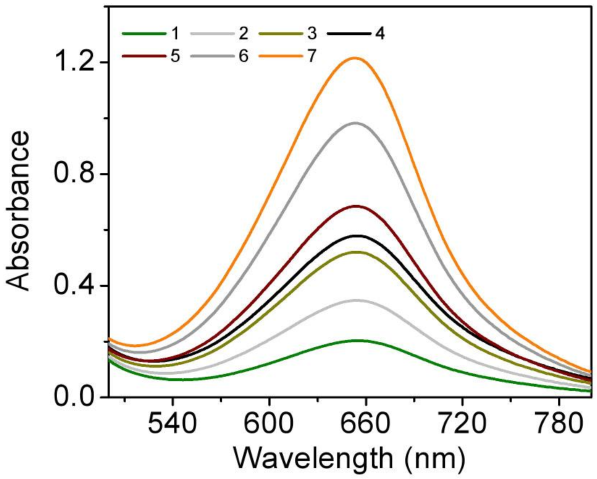

Figure S17. UV–vis spectra of CoP@TiO_2/TP-based Zn-NO_2^- battery for NH_3 generation at different current density, related to Figure 4.

Table S1. Comparison of catalytic performances of CoP@TiO_2/TP with other reported NO_2^-RR electrocatalysts, related to Figure 1.

Catalyst	Electrolyte	FE (%) @potential (V)	NH_3 yield (mmol h^{-1} cm^2)@ potential (V)	Ref.
**CoP@TiO_2/TP**	**0.1 M NaOH (0.1 M NO_2^-)**	**97.01@−0.3**	**0.8496@−0.6**	**This work**
Ru-Cu NW/CF	0.1 M PBS (500 ppm NO_2^-)	94.1@−0.6	0.732@−0.6	Chem. Commun. 58, 52571 (2022)
Ag@NiO/CC	0.1 M NaOH (0.1 M NO_2^-)	97.7@−0.4	0.338@−0.7	J. Colloid Interf. Sci. 623, 513 (2022)
CoP/CC	1 M NaOH (0.002 M NO_2^-)	91.6@−0.3	0.022@−0.3	J. Electroanal. Chem. 910, 116171 (2022)
CoP NA/TM	0.1 M PBS (500 ppm NO_2^-)	90.0 ± 2.3@−0.2	0.133 ± 0.003@−0.2	Nano Res. 15, 972 (2022)
Cobalt-tripeptide complex	1.0 M MOPS (1.0 M NO_2^-)	90 ± 3@−0.7	0.0011@−0.7	J. Am. Chem. Soc. 140, 16888 (2018)
CoB nanoarray	0.2 M Na_2SO_4 (400 ppm NO_2^-)	95.2@−0.5	0.2331@−0.5	Inorg. Chem. Front. 9, 6075 (2022)
TiO_{2-x}	0.1 M NaOH (0.1 M NO_2^-)	92.7@−0.7	0.464@−0.7	Chem. Commun. 58, 3669 (2022)
P-TiO_2	0.1 M NaOH (0.1 M NO_2^-)	90.6@−0.6	0.561@−0.6	J. Mater. Chem. A 10, 23494 (2022)
V-TiO_2	0.1 M NaOH (0.1 M NO_2^-)	93.2@−0.6	0.5408@−0.7	Mater. Today Phys. 30, 100944 (2023)
Cu_3P NA/CF	0.1 M PBS (0.1 M NO_2^-)	91.2 ± 2.5@−0.5	0.0956@−0.5	Green Chem. 23, 5487 (2021)
Ni@JBC	0.1 M NaOH (0.1 M NO_2^-)	83.4@−0.5	0.242@−0.5	Nanoscale 14, 13073 (2022)
Ni@MDC	0.1 M NaOH (0.1 M NO_2^-)	65.4@−0.8	0.371@−0.8	ACS Appl. Nano Mater. 5, 14246 (2022)
Ni-NSA-V_{Ni}	0.2 M Na_2SO_4 (200 ppm NO_2^-)	96.1@−0.54	0.236@−0.54	J. Mater. Chem. A 9, 239 (2021)
FeOOH NTA/CC	0.1 M PBS (0.1 M NO_2^-)	94.7@−1.0	0.702@−1.1	Chem. Commun. 58, 5160 (2022)
